# Cross-Cultural Ethnobotanical Assembly as a New Tool for Understanding Medicinal and Culinary Values–The Genus *Lycium* as A Case Study

**DOI:** 10.3389/fphar.2021.708518

**Published:** 2021-07-16

**Authors:** Ruyu Yao, Michael Heinrich, Jianhe Wei, Peigen Xiao

**Affiliations:** ^1^ Institute of Medicinal Plant Development, Chinese Academy of Medical Sciences and Peking Union Medical College, Beijing, China; ^2^ Research Group “Pharmacognosy and Phytotherapy”, UCL School of Pharmacy, Univ. London, London, United Kingdom

**Keywords:** ethnobotany, ethnopharmacology, Lycium, medicine and food homology, traditional use

## Abstract

Ethnobotanical knowledge is indispensable for the conservation of global biological integrity, and could provide irreplaceable clues for bioprospecting aiming at new food crops and medicines. This biocultural diversity requires a comprehensive documentation of such intellectual knowledge at local levels. However, without systematically capturing the data, those regional records are fragmented and can hardly be used. In this study, we develop a framework to assemble the cross-cultural ethnobotanical knowledge at a genus level, including capturing the species’ diversity and their cultural importance, integrating their traditional uses, and revealing the intercultural relationship of ethnobotanical data quantitatively. Using such a cross-cultural ethnobotanical assembly, the medicinal and culinary values of the genus *Lycium* are evaluated. Simultaneously, the analysis highlights the problems and options for a systematic cross-cultural ethnobotanical knowledge assembly. The framework used here could generate baseline data relevant for conservation and sustainable use of plant diversity as well as for bioprospecting within targeting taxa.

## Introduction

Ethnobotanical research records how indigenous peoples use plants and manage the ecosystem, is indispensable for the conservation of global biological integrity, and could provide essential information on bioprospecting aiming at new food crops and medicines (Hoban et al., 2020; Pei et al., 2020; Ulian et al., 2020; Atanasov et al., 2021). The diversity of regional plants and peoples making their traditional knowledge enormously diverse. Therefore, to achieve a comprehensive documentation of such intellectual knowledge should rely on studies at local level (Gaoue et al., 2017; Teixidor-Toneu et al., 2018). Since both the plant species and their associated traditional knowledge are at risk of being lost, their conservation and sustainable use have become a topic of global concern. At the same time, in recent decades, regional studies have recorded tremendous ethnobotanical knowledge (Borrell et al., 2020; Pei et al., 2020). However, there is a need for capturing that information systematically, since otherwise those regional records are fragmented and can hardly be used.

In genomics, scientists use the strategy of “assembly” to fuse fragmented sequences (reads), which are obtained from sequencing technologies, into longer sequences or even into the level of chromosome or genome, thus the genetic information of an organism can be studied further (Nagarajan and Pop, 2013; Dominguez Del Angel et al., 2018). Borrowing the concept from genomics, we propose to assemble the scattered local ethnobotanical knowledge globally. Considering that the chemical constitutions and usages of plants are often phylogenetically conserved (Gaoue et al., 2017; Garnatje et al., 2017), it is advisable to assemble the ethnobotanical knowledge using a group of closely related taxa (e.g., a genus) as a basic unit. Accordingly, we propose a framework for a systematic understanding on any taxon’s ethnobotanical knowledge cross-culturally, including 1) a documentation-based taxonomical revision, 2) evaluating the traditional uses, 3) evaluating the use values of species, and 4) comparing the ethnobotanical knowledge among cultures. Such an assemblage offers a pragmatic solution to understanding (ethno-) botanical diversity.

The genus *Lycium* is used widely as food and medicine (Yao et al., 2018a), and its global distribution and diverse uses make it an ideal example for such an assembly study. For example, in recent years *L. barbarum* L., the most commonly used species of this genus, has become a globally used commodity China produces 250,000–300,000 tons of dried fruits annually, while the amounts produced in other countries are not well known. Many other *Lycium* spp. are also used regionally as food and medicine (Yao et al., 2018a; Yao et al., 2018b). Therefore, as a case study, the present study applies the proposed framework to this genus of global importance.

## Materials and Methods

### Documentation-Based Taxonomic Revision

Accepted species were extracted from the global important plant databases including Catalogue of Life (COL) (Roskov et al., 2020), Plants of the World Online (POWO) (POWO, 2019), World Flora Online (WFO) (WFO, 2021), and the Plant List (TPL) (The Plant List, 2013). GBIF (gbif.org) is among the global important plant databases, since it is a secondary database, its constitute datasets have heavy overlaps with the above (e.g., COL), the current research does not include it. A comparison table was created using the above species lists (see Supplementary Material), which was used to generate a revised species list as follows:

(1) Species were classed as taxonomically valid (i.e., “accepted”), if they were accepted in all the three databases;
(2) Varieties and hybrids were removed;
(3) Species names accepted only in one or two of the databases were cross-checked using primary taxonomic sources and regional floras, as well as virtual specimens of the disputed species;
(4) debatable names were accepted based on the International Code of Botanical Nomenclature, with consideration of the information collected in step (3);
(5) The status of synonyms was ascertained based on the above evidences, and confirmed synonyms were removed.

Thus, the accept species together formed a revised species list. An UpSet graph was then produced.

### Evaluating the Traditional Uses

The traditional uses of *Lycium* spp. as food, medicine and in rituals were extracted from journal articles, ethnobotanical monographs, online ethnobotanical databases. The medical usages were categorized following International Classification of Diseases 11th Revision (ICD-11) (World Health Organization, 2020), and the traditional medical indications were categorized according to chapter two6 of ICD-11, with extra classifications when necessary. The culture backgrounds were extracted from the source literatures, and, if this was not stated in the reference, their languages and language families were searched with WALS (The World Atlas of Language Structures) (Dryer and Haspelmath, 2013) and international standards of ISO 639-2 (Library of Congress, 2017) and ISO 639-5 (Library of Congress, 2013). A 3-letter code of the language family is used as an identity of the cultural background. Languages of jpn and kor, belonging to the family of tut, were treated as independent cultures considering they have succeeded the Chinese (which is belonging to sit) knowledge of using *Lycium* directly. Note that if an abbreviation was not included the above references, a 3-letter code was then created, such as languages in Chile and Argentina, Austro-Asiatic (Vietnamese), Matacoan-Chorote, Aboriginal (Australian), Keresan-Acoma, Guaicuruan, Muskogean, and Kiowa-Tanoan [for a complete check list of language (families) and their codes see Supplementary Material]. Accordingly, a matrix including all species and their corresponding usages and cultural backgrounds was built and presented as a heatmap using TBtools (Chen et al., 2020).

The sequence of the granule-bound starch synthase gene (GBSSI) was used as a DNA barcode to show the phylogenetic relationship amongst the selected *Lycium* spp. 72 *Lycium* spp. were selected, which included 34 of the 36 traditional used species, with *Nolana werdermannii* as the outgroup, and their accession numbers of NCBI were listed in Supplementary Material. The phylogenetic tree was constructed using the statistical method of Maximum Likelihood, and tested by bootstrap method with 500 replications. The phylogenetic tree was then fitted to the above heatmap.

In order to evaluate the potential of *Lycium* for any traditional uses, we used a modified Fidelity Level (FL) Index (Friedman et al., 1986), since we use it for the evaluation of a genus instead of a species. FL was calculated as follows:

$$FL_{u}=\frac{R_{u}}{N}$$

Where: Fl_u_ is the FL of a specific use, while R_u_ is the number of reports for a specific use, and N is the number of cultures with a recorded use.

The FL_u_ values were then appended to the above heatmap.

### Evaluating the Use Values of Species

Cultural Importance Index (CII) (Tardío and Pardo-de-Santayana, 2008), Relative Frequency of Citation (RFC) (Tardío and Pardo-de-Santayana, 2008), and Use Value (UV) (Rossato et al., 1999) were used to evaluate the importance of every species, with adaptions. The above indices were calculated as follows.

$$CII_{s}=\frac{R_{uc.s}}{N}$$

Where: CII_s_ is the CII of a specific species S; R_uc. s_ is the number of reports for a use category (here the categories of medicinal use, food use, and ritual use are calculated respectively) of the species; N is the number of informants (here it refers to number of all the cultures).

$$RFC_{s}=\frac{N_{s}}{N}$$

Where: RFC_s_ is the RFC of a specific species S; N_s_ is the number of informants who report the use of species S; N is the number of informants (here it refers to number of all the cultures).

$$UV_{c.s}=\frac{R_{c.s}}{N_{s}}$$

Where: UV_c.s_ is the UV of a specific species S for the use category C; Rs is the number of use reports of species S; N_s_ is the number of informants who report the use of species S. Considering there was no subgroup for food or ritual uses, only the UVs of medicinal use were calculated.

### Comparing the Ethnobotanical Knowledge Among Cultures

Jaccard Index (JI), an index widely used in many fields of science was used to study the ethnobotanical knowledge based on cultural backgrounds (Wang and Wang, 2017). In this case, JI was employed to compare the species used by peoples of different language families, with adaptions. The data on uses was categorized by the language families of people, and then, pairwise comparisons were calculated according to the following formula.

$$JI_{AB}=\frac{N_{A\cap B}}{N_{A\cup B}}\;\times 100$$

Where: JI_AB_ is the JI between the two cultural backgrounds of A and B; N_A∪B_ is the number of union species used in A and B; N_A∩B_ is the number of intersection species used in A and B.

The results were then complied into a correction matrix, which was further presented as a correction heatmap.

## Results and Discussion

### Documentation-Based Species List Revision

**FIGURE 1:**
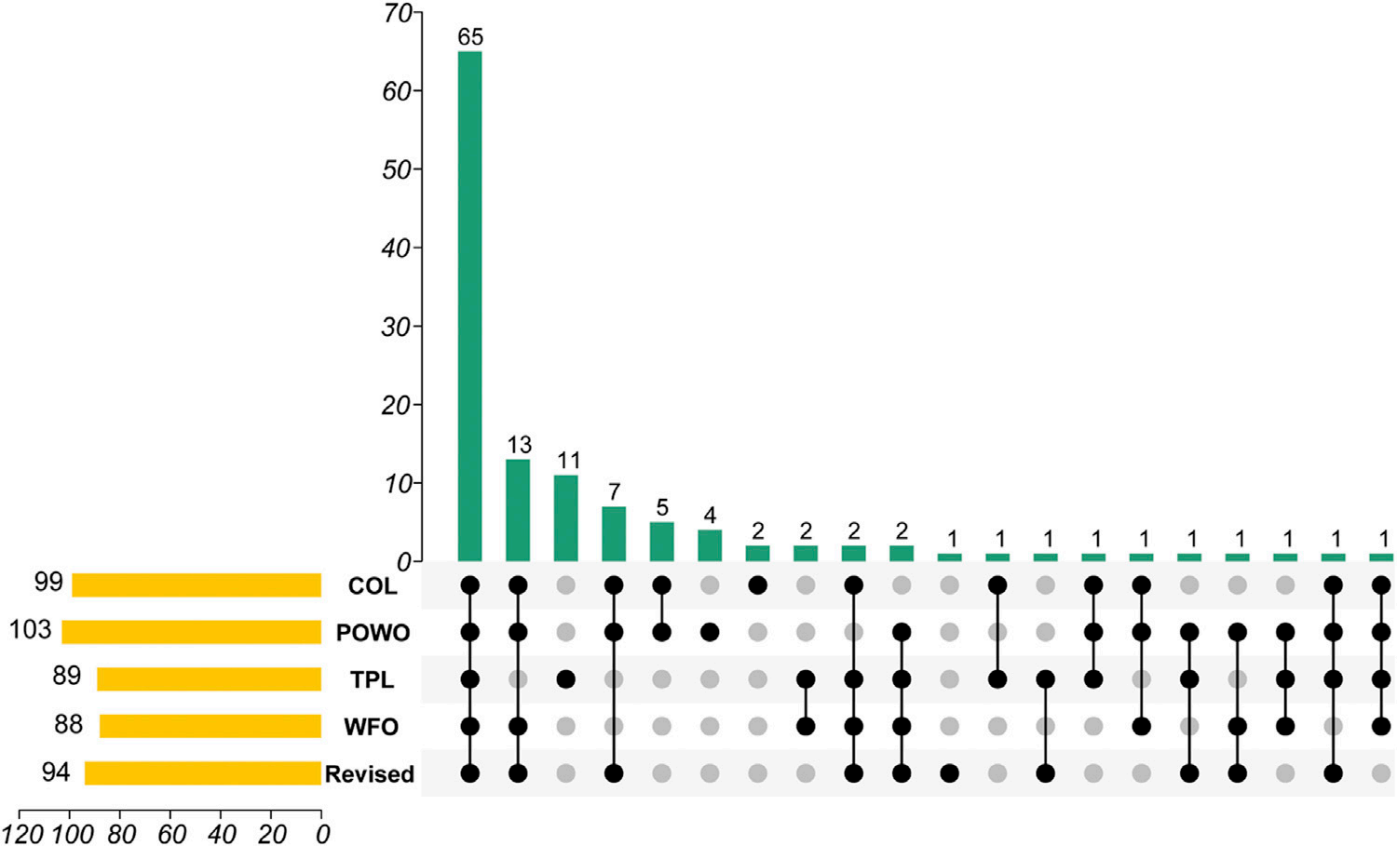
An UpSet graph of *Lycium* spp. among databases covering species. Data source: Catalogue of Life (COL), Plants of the World Online (POWO), World Flora Online (WFO), and the Plant List (TPL). The bottom part indicates the coverage of individual species in the respective databases. For example, the second vertical bar indicates that there are 13 species listed in four (COL, POWO, WFO, and Revised) of the five databases, excluding TPL, while the third vertical bar indicates eleven are only found in TPL.

Similar to a defined sequence of a gene or group of genes, the valid botanical name is the identifier of a plant, without which even the entity of the traditional knowledge is ambiguous (Bennett and Balick, 2014; Rivera et al., 2014; Yao et al., 2021). Considering its importance to the biodiversity conservation, “an online flora of all known plants” was set as the primary target of the Global Strategy for Plant Conservation (GSPC) (Jackson and Miller, 2015). Currently, Catalogue of Life (COL), Plants of the World Online (POWO), World Flora Online (WFO) have been widely recognized as the important global nomenclatural standards for species names (POWO, 2019; Roskov et al., 2020; WFO, 2021). Moreover, the Plant List (TPL not updated since 2013) was used as the starting point for the Taxonomic Backbone of the WFO (The Plant List, 2013). Tropicos provides an index of plant names and references (Missouri Botanical Garden, 2021). Clearly, regional floras provide important baseline data for the species description and identification. Using on-line herbaria and libraries, the type specimens and the original published literature of controversial names were traced, based on which a corrected list of the genus *Lycium* was constructed. A comparison of *Lycium* species lists of COL, POWO, WFO, TPL, and the revised list is provided in the Supplementary Material. It is found that the lists of *Lycium* species are markedly different among those global databases (Figure 1). The numbers of accepted scientific names included in those databases range from 88 to 103, with only 65 in common to all five sources. Eleven names are only indexed in TPL while 13 spp. are included in the other four, showing the progress of revision work since 2013. The revised species list we developed has corrected several misused names in the present international databases.

### Assembly of Traditional Uses

**FIGURE 2:**
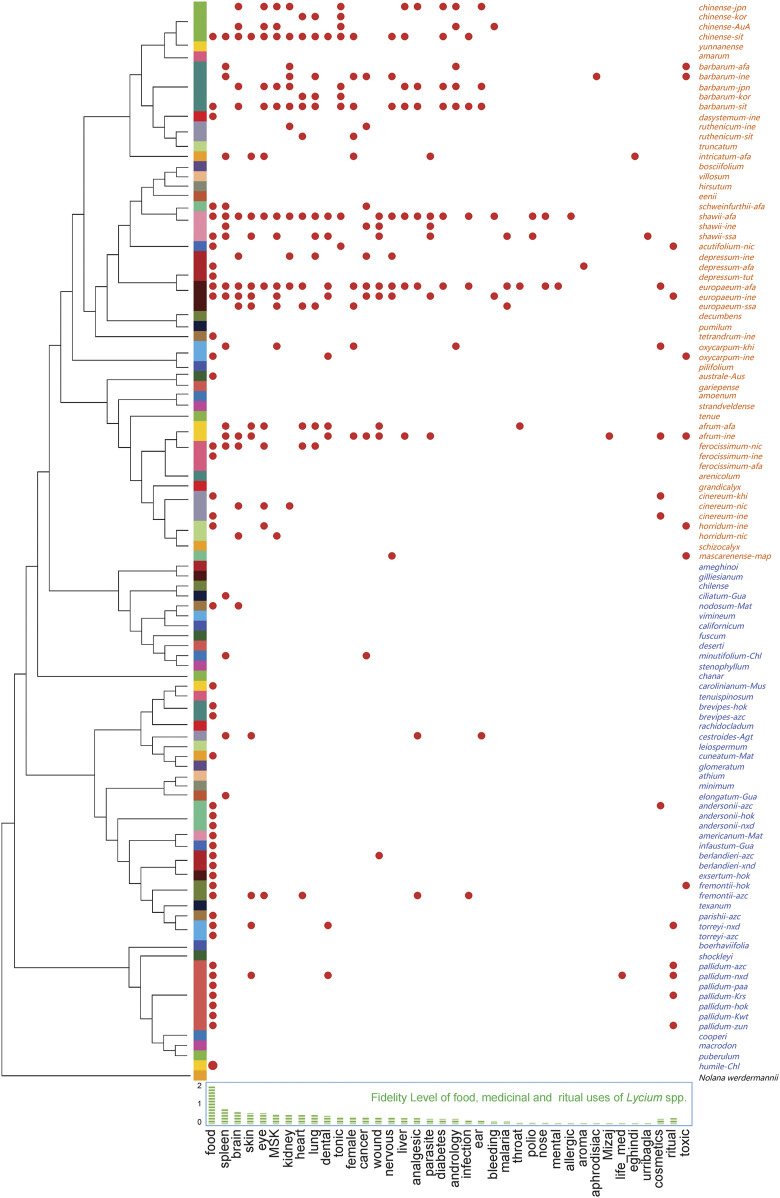
The food, medicine, and ritual use of *Lycium* spp., with phylogenetic relationship reconstructed by sequences of the granule-bound starch synthase gene (GBSSI), and the Fidelity Level of the uses. Note that items are named as “epitheton-language family” (the Old World species are in orange and the New World species are in blue), and language families are obtained by WALS (The World Atlas of Language Structures) and international standards of ISO 639-2 and ISO 639-5; GBSSI sequences were retrieved from NCBI. Lists of language families, IDs of selected sequences, and references of the plant uses are presented in the Supplementary Material.

The species used and their usages were extracted from journal articles, ethnobotanical monographs, and online ethnobotanical databases. The medical usages were categorized following International Classification of Diseases 11th Revision (ICD-11) with extra classifications when necessary. All species with their corresponding usages and cultural backgrounds (Figure 2,) include the cultural backgrounds represented by the language families (see Supplementary Material). 36 *Lycium* species are recorded to be used as food or medicine by peoples of diverse cultural backgrounds. Of these, 28 species are used for culinary purposes, with a Fidelity Level (FL) of as high as 1.84 (Figure 2, bottom). As for medicinal uses, *Lycium* spp. are frequently applied to treatments for diseases of the spleen, brain, skin, eye, musculoskeletal (MSK), kidney, heart, lung, dental, among others, with FL ranging from 0.72 to 0.40, indicating their high potential medicinal values for related drugs. Besides, solitary medical uses, such as urribaqla, eghindi, Mizaj, etc., are found in local records only, many of which are panacea-like with special conceptual indications and with very low FL. Moreover, *Lycium* spp. are also reported as cosmetics (FL = 0.24) and for ritual uses (FL = 0.28). While there are a variety of useful properties, six species are reported to be toxic in specific conditions. This ethnobotanical knowledge assemblage provides useful clues for finding new food sources and new drugs from the genus. Bioprospecting aiming at new drugs from plants requires specific verifications at different stages, when the chemical composition of a plant is clear, its traditional uses could be verified preliminarily with network pharmacology (Lu et al., 2020).

Using the sequence of the granule-bound starch synthase gene (GBSSI) as a DNA barcode, the phylogenetic relationship of the selected *Lycium* spp. is presented with a biogeographic pattern: The New World species are separated from those of the Old World, while those eastern Asian species form a sub-cluster within the cluster of Old World species (Figure 2, left side). Apparently, the usages of the Old World species are more diverse than those of the New World. Hot clades of culinary or medicinal used species are found in the clusters of eastern Asian species, western Asian and Mediterranean species, and a cluster compromises of several South and North American species.

### Quantitative Evaluation of Medicinal and Culinary Values

**FIGURE 3:**
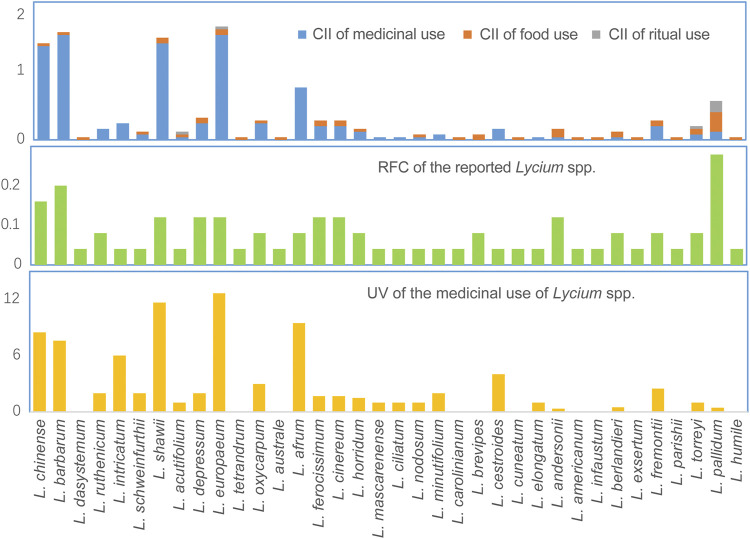
Cultural Importance Index (CII) and Relative Frequency of Citation (RFC) of *Lycium* spp., and Useful Value (UV) of their medicinal uses.

The cultural importance of individual species can be indicated by their Cultural Importance Index (CII), Relative Frequency of Citation (RFC), and Useful Value (UV), as are shown in Figure 3. Five species of the Old World, *viz. L. chinense, L. barbarum, L. shawii, L. europaeum,* and *L. afrum*, have considerable importance in medical uses, with CII ranging from 1.52 to 0.76, and their significant medical values are also supported by the high RFC and UV. In contrast, the species of New World are more commonly used as food, especially *L. pallidum* and *L. andersonii* with CIIs of 0.28 and 0.12, respectively. Moreover, using diverse processing methods, fruits, shoots, and leaves of *Lycium* spp. are prepared into the forms of syrup, beverage, delicacy, mixed paste, porridge, and so on, and consuming the fresh fruits is also common. Furthermore, four species are found to be used for ritual purposes, which emphases the important role of *Lycium* in indigenous peoples’ daily lives.

The “medicine and food homology” is commonly seen in the traditional uses of plants (Jennings et al., 2015; Liu, 2018), which indicates the commonality of plants’ medicinal and culinary values. The above analyses give a quantitative overview on the medicinal and food uses of targeting species at a global level, thus to make the ethnobotanical knowledge available for further research.

### Cross-Cultural Comparison of the Traditional Uses

**FIGURE 4:**
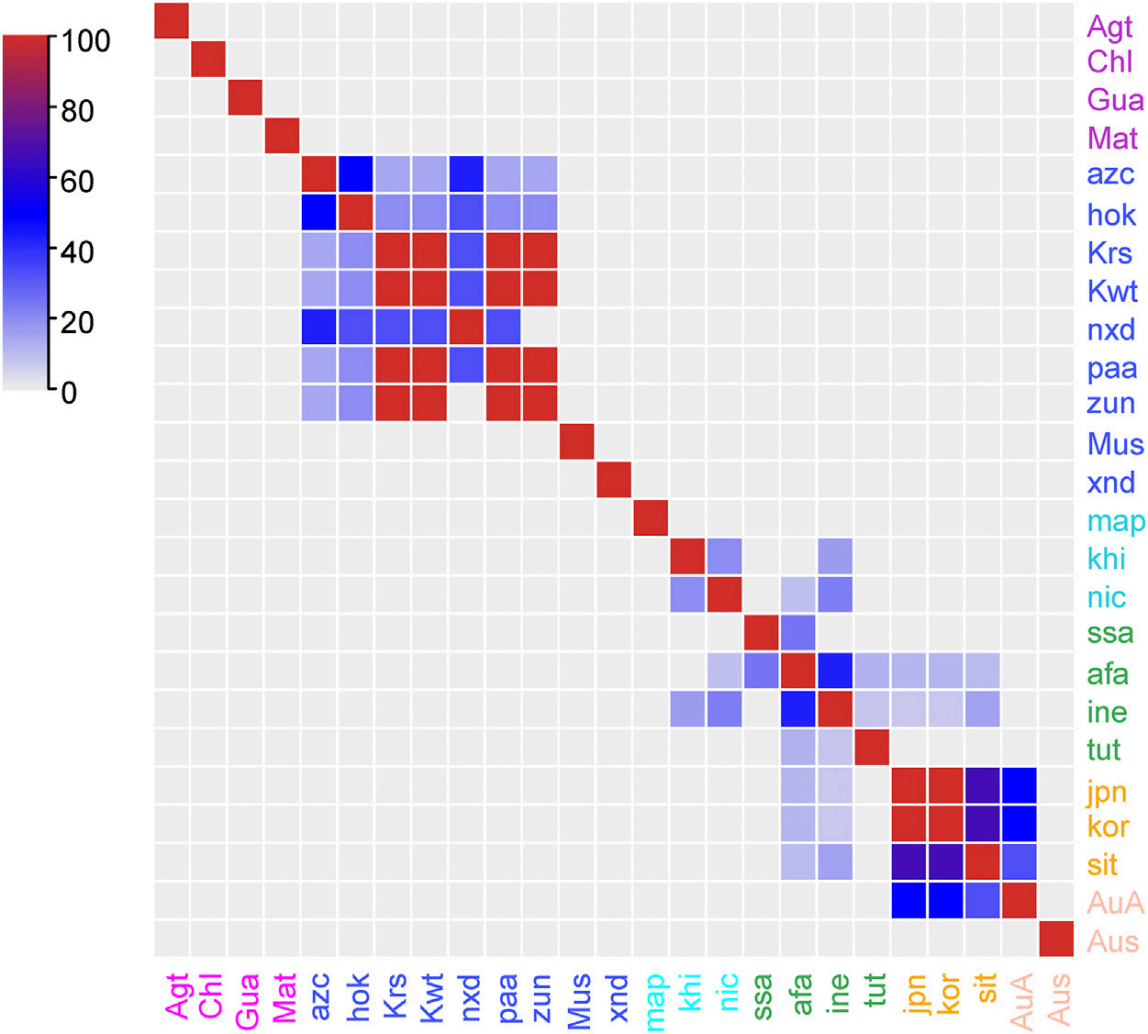
Pairwise comparison of the Jaccard Index (JI) between ethnobotanical knowledge on *Lycium* spp. of different cultures as represented by language families.

Since the traditional use of plants is impacted by the cultural background, which can be represented by the language family of people, here we use language families of people as indicators of their cultural backgrounds. Jaccard Index (JI), an index commonly used in ecologic studies, is applied to compare the use similarity among cultures. As is shown in Figure 4, the use in four cultures of South America (Agt, Chl, Gua, and Mat), two cultures of North America (Mus and xnd), one culture of Africa (map), and one culture of Australia (Aus) are isolated. The discrete distribution of the species and the lack of communication among cultures may lead to the isolated knowledge on uses (Quave and Pieroni, 2015). High JI regions are found among seven cultures of North America, as well as 10 cultures of Africa and Eurasia. North American cultures are geographically contiguous, providing the opportunities for sharing plant resources and knowledge. The southern African cultures are less similar compared to others but with significant similarity with their close neighbours. The culture of ine (Indo-European) and afa (Afro-Asiatic) have broad link with many other cultures, including those of long geographic distances. These two language families cover large geographic region and the splendid ancient civilizations of the world. In the early times, people of ine and afa were the frequent travellers especially for business across the continents, and played important roles in cross-cultural communications.

A verifiable source of evidence is an ancient Islamic-oriented herbal, *Huihui Yaofang*, which was written in Chinese in the era of Genghis Khan, and it is still being used by people of western China (Kong and Chen, 1996). Moreover, cultures of sit (Sino-Tibetan), kor (Korean), jpn (Japan), and AuA (Austro-Asiatic) show considerable similarity. Except for their geographic linkage, the cross-cultural communication among them has contributed to knowledge sharing, which are supported by the historical herbals and traditional phytotherapies communicated for over one millennium, such as the Chinese-derived Dongui Bogam and Kampo. Consequently, as one would expect (Heinrich et al., 2005), the similarity of ethnobotanical knowledge reflects the cross-cultural communication, which could be understood using a JI analysis.

The conservation and sustainable use of plant resources and their associated traditional knowledge have given rise to global concerns. Considering their importance in biodiversity conservation, “an online flora of all known plants” was set as the primary target of the GSPC. Simultaneously, indigenous and local knowledge of plant species is also been stressed in the Aichi Biodiversity Targets (Convention on Biological Diversity, 2012a; Convention on Biological Diversity, 2012b; Jackson and Miller, 2015). The framework presented here allows for the plant name revision and ethnobotanical knowledge organization of any taxon, which is a pragmatic tool for the implementation of GSPC and the Aichi Targets. While emphasizing the culinary and medicinal assessment, it could provide irreplaceable clues for bioprospecting aiming at new foods and medicines.

## Conclusion

Ethnobotanical knowledge is critical for the conservation and utilization of local plants. Although plant resources and their popular and traditional usages in different geographic regions and by people with different cultural backgrounds are recorded increasingly, such fragmented catalogues have limited scientific value without systematic system to capture and compare data. This problem is especially urgent since the plant species and their associated traditional knowledge are at risk of being lost. Therefore, borrowing the concept of assemblage from genomics, we propose a framework for a systematic understanding on any taxon’s ethnobotanical knowledge. The assembly of the genus *Lycium* indicates the requirement for a documentation-based taxonomic revision to current updated international species checklists. Additionally, the quantitative evaluations highlight the medicinal and culinary value of *Lycium* spp., while the corresponding species are also clarified, which contributes a deeper understanding into the cultural importance of this genus. It can be applied to groups of related taxa like genera or groups of related genera. Next a larger picture at a family level and beyond can be built up. Moreover, the comparisons among cultures support that the cross-cultural communications lead to the sharing of ethnobotanical knowledge. This paper offers a perspective and a framework for achieving this and the concept will need to be refined further using other case studies The extending application of this framework will facilitate a better appreciation of plant biodiversity, and will be helpful for the conservation and sustainable utilization of local ethnobotanical knowledge.

## Data Availability

The datasets presented in this study can be found in online repositories. The names of the repository/repositories and accession number(s) can be found in the article/Supplementary Material.
